# Localized periodontitis and kidney function for the risk of proteinuria in young adults in the CHIEF oral health study

**DOI:** 10.1038/s41598-022-23843-0

**Published:** 2022-11-08

**Authors:** Kun-Zhe Tsai, Pang-Yen Liu, Tsung-Jui Wu, Chia-Hao Fan, Wan-Chien Cheng, Ren-Yeong Huang, Gen-Min Lin

**Affiliations:** 1 Department of Medicine, Hualien Armed Forces General Hospital, No. 163, Jiali Rd., Xincheng Township, Hualien City, 97144 Taiwan; 2 Department of Stomatology of Periodontology, Mackay Memorial Hospital, Taipei, Taiwan; 3 Departments of Dentistry, National Defense Medical Center, Tri-Service General Hospital, Taipei, Taiwan; 4 National Defense Medical Center, Graduate Institute of Dental Science, Taipei, Taiwan; 5 Department of Internal Medicine, National Defense Medical Center, Tri-Service General Hospital, Taipei, Taiwan; 6 Department of Nursing, Hualien Armed Forces General Hospital, Hualien, Taiwan

**Keywords:** Diseases, Nephrology, Risk factors

## Abstract

This study aimed to investigate the association of localized periodontitis with proteinuria in 1281 military young adults in Taiwan. Localized periodontitis was classified as Healthy/Stage I (N = 928) or Stage II/III (N = 353). Stage 2 chronic kidney disease (CKD) was defined as an estimated glomerular filtration rate (eGFR) of 60–89 mL/min/1.73 m^2^. Proteinuria was defined as protein levels of 2+ or 3+ on the dipstick test. Multiple logistic regression analysis with adjustments for age, sex, body mass index, remaining teeth number and other potential covariates were used to determine the association between localized Stage II/III periodontitis and dipstick proteinuria in patients with and without CKD. Localized stage II/III periodontitis was associated with a higher risk of dipstick proteinuria [odds ratio (OR) and 95% confidence interval: 1.89 (1.04–3.42)], but not with stage 2 CKD. However, the association between localized stage II/III periodontitis and dipstick proteinuria was observed only in patients with stage 2 CKD [OR: 3.80 (1.56–9.27)], while the association was null in participants without stage 2 CKD [OR: 1.02 (0.42–2.45)]. Our findings suggest that among young adults, especially those with a mildly impaired eGFR, localized periodontitis might contribute to acute or chronic kidney injury, which manifests as proteinuria.

## Introduction

Periodontitis is a polymicrobial disease associated with several pathogenic bacteria in the subgingival biofilm and an imbalance in the microbial ecological environment in the mouth^1^. The effects of periodontitis are not limited to the oral cavity, and may have a negative impact on systemic health. Over the past few decades, substantial evidence has accumulated on the links between periodontitis and many extra-oral systemic disorders, e.g. obesity^2^, metabolic syndrome^3^, and hepatitis^4^. Chronic low-grade inflammation is a common pathogenic denominator of periodontitis and is associated with systemic disorders^5^.

Chronic kidney disease (CKD), characterized by a reduced estimated glomerular filtration rate (eGFR) and the presence of microalbuminuria or proteinuria, is a public health problem worldwide, with an estimated global prevalence of 13.4%^6^. Patients with initial-stage CKD are usually asymptomatic for many years and present with typical complications of renal dysfunction only in more advanced stages^7^. Periodontitis is a novel and potentially modifiable risk factor for advanced CKD^8^. Periodontal pathogens may migrate systemically via the bloodstream and affect the endothelial function of the nephrons^9,10^. Additionally, periodontitis can alter systemic homeostasis through the release of endotoxins and inflammatory cytokines^11^.

A large body of evidence supports the relationship between generalized periodontitis and severe CKD ≥ Stage 3 in middle-aged and older adults^12–15^. Since the CKD stage and incidence of periodontitis are relatively low in young adults, there have been few studies regarding this association in this population. Therefore, this study aimed to investigate the association between localized periodontitis and acute or chronic kidney injury in young adults with and without initial CKD.

## Methods

### Study design and population

The study population was selected from participants of the Cardiorespiratory Fitness and Health in Eastern Armed Forces (CHIEF) oral-health study^2–4,16,17^, which was performed at the Hualien Armed Forces General Hospital in Taiwan, between 2018 and 2021. This study was reviewed and approved by the institutional review board of Mennonite Christian Hospital (No. 16-05-008) in Hualien City, Taiwan, and written informed consent was obtained from all participants. All participants underwent an annual military health examination including oral health screening and other laboratory tests. Each participant completed a self-administered questionnaire evaluating alcohol consumption, smoking status, and educational level. Alcohol intake and tobacco smoking status were categorized as never, former, or current. The degree of education was defined according to the highest school grade (senior high school, college or university, and postgraduate degree). The present study was conducted in accordance with the tenets of the Declaration of Helsinki, 1975, as revised in 2013.

### Physical and laboratory measures

Anthropometric parameters, including waist circumference, body height, and body weight, were measured in the standing position. Body mass index was calculated as body weight (kg) divided by the square of body height (m). Blood pressure (BP) was measured on the right upper arm using an automatic oscillometric machine (Parama-Tech Co Ltd, Fukuoka, Japan). Mean BP was calculated as 1/3 x (2 × systolic BP + 1 × diastolic BP). After a 24-h overnight fast, blood samples were collected from the antecubital vein to measure the total cholesterol, high-density lipoprotein cholesterol (HDL), triglycerides, fasting glucose, creatinine, and blood urea nitrogen levels.

The International Diabetes Federation definition^18^ identified five main cardiometabolic risk factors for vascular diseases and CKD: (1). low serum HDL, (2). high serum triglycerides or with lipid-lowering therapy, (3) hyperglycemia or with antidiabetic therapy, (4) elevated BP or with anti-hypertensive treatment, and (5) central obesity defined as waist circumference ≥ 90 cm for adult males.

### Periodontal assessments

A comprehensive periodontal examination was performed to evaluate the severity of the periodontitis. Third molars and impacted teeth were excluded from the present study. Periodontal status was categorized as Healthy/Stage I or Stage II/III periodontitis according to the 2017 workshop of the American Academy of Periodontology (AAP)/European Federation of Periodontology (EFP)^19^,. As our population included young adults, periodontitis was localized (< 30% of teeth involved)^19^ in most participants. Periodontal parameters, e.g. probing depth (PD), clinical attachment loss (CAL) and bleeding on probing (BOP) were reported.

### Assessment of kidney function and proteinuria

eGFR was calculated using the abbreviated Modification of Diet in Renal Disease formula^20^ using the following equation: eGFR = 186 × serum creatinine (mg⁄dL)^−1.154^ × age (years)^−0.203^. Renal function was graded based on the eGFR level; normal renal function was defined as an eGFR ≥ 90 mL/min/1.73 m^2^ and Stage 2 CKD was defined as an eGFR of 60–89 mL/min/1.73 m^2^^20^.

Random urine samples were obtained using the clean-catch technique in a sterile container prior to venipuncture for blood sampling in the morning to avoid stress confounding the interpretation of proteinuria. Urine proteins were graded using AUTION Dipsticks (ARKRAY. Inc., Shiga, Japan) using an AX-4030 urine-chemistry analyzer (ARKRAY Inc, Shiga, Japan). Urine protein concentrations of 30–99 mg/dL, 100–299 mg/dL, 300–999 mg/dL, and ≥ 1000 mg/dL were labeled as “1 + ,” “2 + ,” “3 + ” and “4 + ,” respectively, based on the clinical classifications recommended by the National Kidney Foundation^21^. The intra-assay coefficient of variation for proteinuria grading was 6.08%.

### Statistical analysis

The clinical profiles of participants with normal renal function and those with Stage 2 CKD are presented as mean ± standard deviation (SD) for continuous data and as numbers (%) for categorical data. Continuous variables were compared using one-way analysis of variance, and categorical variables were compared using the X^2^ test. Multiple logistic regression analysis was used to determine ORs and 95% CIs of localized Stage II/III periodontitis with Stage 2 CKD and dipstick proteinuria, separately. Multiple linear regression analysis for the associations of eGFR and grades of dipstick proteinuria (absence, 1+ , 2+ , 3+ and 4+) with stages of localized periodontitis (healthy, I, II and III), PD and CAL were also performed, separately. In addition, multiple logistic regression analysis was used to determine the association between Stage II/III periodontitis and dipstick proteinuria in participants with and without Stage 2 CKD. Covariates were adjusted stepwise in three models. In Model 1, age, sex, alcohol intake, smoking, and education level were adjusted. In Model 2, Model-1 covariates plus BMI, mean BP, fasting glucose, total cholesterol, and serum triglycerides were adjusted. In Model 3, Model-2 covariates plus the number of remaining teeth were adjusted. Statistical significance was set at *p* < 0.05. The SPSS statistical software was used for all the statistical analyses (IBM SPSS Statistics for Windows, Version 25.0; IBM Corp., Armonk, NY).

## Results

**Figure 1:**
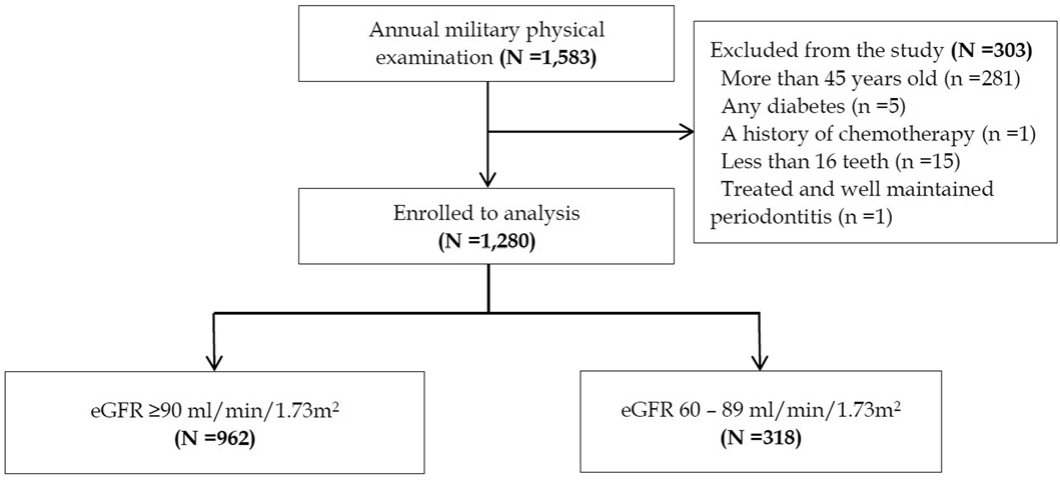
Flow diagram for selection of participants and design of the CHIEF oral health study.

A total of 1583 participants were enrolled in the present study. Of these, 303 participants were excluded, including patients with age > 45 years (N = 281), Type 1 or Type 2 diabetes (N = 5), a history of chemotherapy (N = 1), < 16 teeth (N = 15), and treated and well maintained periodontitis (N = 1). The final sample consisted of 1280 young men and women, aged 18–45 years. The flow diagram for the selection of study participants is shown in Fig. 1.

Of the 1280 study participants, 962 (75.2%) had normal renal function, and 318 (24.8%) had Stage 2 CKD, and no one had Stage 3 or Stage 4 CKD (Table 1). Those with Stage 2 CKD were relatively older and had higher levels of several metabolic risk factors, e.g. body mass index, BP, total cholesterol, and serum triglycerides. However, there were no differences in the HDL or fasting glucose levels. Regarding the oral condition, there were relatively fewer remaining teeth in patients with Stage 2 CKD. However, there were no differences in prevalence of bleeding on probing and periodontitis-related indexes (Table 1).

**Table 1:** Demographic and clinical characteristics of the study population (N = 1280).

	eGFR ≥ 90 mL/min/1.73 m^2^ (N = 962)	eGFR 60–89 mL/min/1.73 m^2^ (N = 318)	*p*-value
Age, years	29.60 ± 6.03	32.43 ± 5.17	< 0.001
Sex, male (%)	832 (86.5)	296 (93.1)	0.001
Smoking, active (%)	173 (18.0)	64 (20.1)	0.39
Alcohol intake, active (%)	151 (15.7)	53 (16.7)	0.68
**Education level**			
Up to senior high school	336 (34.9)	57 (17.9)	< 0.001
College/University degree	609 (63.3)	251 (78.9)	
Postgraduate degree	17 (1.8)	10 (3.1)	
Body mass index, kg/m^2^	25.53 ± 3.87	26.34 ± 3.39	0.001
Systolic blood pressure, mmHg	121.18 ± 12.65	123.50 ± 11.64	0.004
Diastolic blood pressure, mmHg	73.48 ± 10.28	75.24 ± 10.11	0.008
Mean blood pressure, mmHg	105.27 ± 11.22	107.07 ± 12.11	0.01
Total cholesterol, mg/dl	178.67 ± 32.84	191.46 ± 40.48	< 0.001
High-density lipoprotein, mg/dl	49.23 ± 10.61	48.68 ± 11.27	0.42
Serum triglycerides, mg/dl	124.73 ± 100.80	142.47 ± 98.01	0.006
Fasting glucose, mg/dl	92.13 ± 11.08	92.88 ± 10.50	0.28
**Serum creatinine, mg/dl**	0.87 ± 0.11	1.08 ± 0.09	< 0.001
(Range: min–max)	(0.44 – 1.10)	(0.77 – 1.44)	
**eGFR, ml/min/1.73m**^**2**^	107.61 ± 14.34	82.45 ± 5.76	< 0.001
(Range: min–max)	(90.0 – 186.7)	(60.8 – 89.9)	
Blood urea nitrogen, mg/dl	12.58 ± 2.94	13.99 ± 2.95	< 0.001
**Proteinuria (%)**			
1+	378 (39.4)	135 (42.6)	< 0.001
2+	22 (2.3)	15 (4.7)	
3+	6 (0.6)	10 (3.2)	
4+	2 (0.2)	0 (0.0)	
Remaining teeth	27.25 ± 1.39	27.03 ± 1.54	0.02
**Localized periodontitis, (%)**	477 (49.6)	191 (60.1)	0.001
Stage I	223 (23.2)	92 (28.9)	0.03
Stage II	114 (11.9)	43 (13.5)	
Stage III	140 (14.6)	56 (17.6)	
BOP (%)	20.25 ± 9.66	27.81 ± 10.64	0.10
**Probing depth, PD (mm)**	2.54 ± 0.93	2.61 ± 0.88	0.22
% of teeth with PD ≥ 6 mm	0.65 ± 0.49	0.77 ± 0.15	0.95
**Clinical attachment loss, CAL (mm)**	2.68 ± 0.91	2.67 ± 0.78	0.54
% of teeth with CAL ≥ 5 mm	0.89 ± 0.53	1.20 ± 0.47	0.98

Continuous variables are expressed as mean ± standard deviation (SD), and categorical variables as N (%).

*eGFR* estimated glomerular filtration rate, *BOP* bleeding on probing.

Table 2 shows the results of multiple logistic regression analysis for the association of dipstick proteinuria and Stage 2 CKD with localized periodontitis in all participants. There was no association between localized periodontitis and Stage 2 CKD in Models 1–3. In contrast, localized periodontitis was associated with dipstick proteinuria in Model 1 (OR, 1.80; 95% CI, 1.00–3.23), Model 2 (OR, 1.90; 95% CI, 1.05–3.43), and Model 3 (OR, 1.89; 95% CI, 1.04–3.42).

**Table 2:** Multivariable logistic regression analysis for stage 2 chronic kidney disease and proteinuria with localized periodontitis in young adults.

	Model 1			Model 2			Model 3		
	OR	95% CI	*p*-value	OR	95% CI	*p*-value	OR	95% CI	*p*-value
eGFR: 60–89 mL/min/1.73 m^2^	1.11	0.83–1.48	0.47	1.07	0.80–1.44	0.64	1.07	0.80–1.44	0.64
Dipstick proteinuria 2+ and 3+	1.80	1.00–3.23	0.04	1.90	1.05–3.43	0.03	1.89	1.04–3.42	0.03

Data are presented as odds ratios (OR) and 95% confidence intervals (CI) using multiple logistic regression analysis models.

Model 1: age, sex, alcohol intake, smoking and education level adjustments.

Model 2: age, sex, alcohol intake, smoking, education level, BMI, mean blood pressure, fasting glucose, total cholesterol and serum triglycerides.

Model 3: age, sex, alcohol intake, smoking, education level, BMI, mean blood pressure, fasting glucose, total cholesterol and serum triglycerides and remaining teeth.

*CKD* chronic kidney disease.

Supplementary Table shows the results of multiple linear regression analysis for the associations of eGFR levels and grades of dipstick proteinuria with stages of localized periodontitis, PD and CAL. The results were consistent with Table 2 that there were no associations between levels of eGFR and stages of localized periodontitis in Models 1–3. The associations of grades of dipstick proteinuria with stages of localized periodontitis were marginally significant in Models 1–3 (β = 0.03, p = 0.07, 0.06 and 0.07, respectively). With regard to other periodontal parameters, there were no associations of eGFR and dipstick proteinuria grades with PD and CAL, respectively in models 1–3.

Table 3 shows the results of multiple logistic regression analysis for dipstick proteinuria with localized periodontitis in patients with and without Stage 2 CKD. There was no association between localized periodontitis and dipstick proteinuria in patients without Stage 2 CKD in Models 1–3. However, localized periodontitis was associated with dipstick proteinuria in participants with Stage 2 CKD in Model 1 (OR, 3.56; 95% CI, 1.48–8.60), Model 2 (OR, 3.80; 95% CI, 1.56–9.25), and Model 3 (OR, 3.80; 95% CI, 1.56–9.27).

**Table 3:** Multivariable logistic regression analysis for the association of localized periodontitis with proteinuria in young adults with and without early chronic kidney disease.

eGFR, mL/min/1.73m^2^	Prevalence of dipstick	Model 1			Model 2			Model 3		
	proteinuria 2+ or 3+	OR	95% CI	*p*-value	OR	95% CI	*p*-value	OR	95% CI	*p*-value
≥ 90	3.1% (30/962)	0.99	0.42–2.34	0.97	1.02	0.43–2.44	0.96	1.02	0.42–2.45	0.96
60–89	7.9% (25/318)	3.56	1.48–8.60	0.005	3.80	1.56–9.25	0.003	3.80	1.56–9.27	0.003

Data are presented as odds ratios (OR) and 95% confidence intervals (CI) using multiple logistic regression analysis models.

Model 1: age, sex, alcohol intake, smoking and education level adjustments.

Model 2: age, sex, alcohol intake, smoking, education level, BMI, mean blood pressure, fasting glucose, total cholesterol and serum triglycerides.

Model 3: age, sex, alcohol intake, smoking, education level, BMI, mean blood pressure, fasting glucose, total cholesterol and serum triglycerides and remaining teeth.

*eGFR* estimated glomerular filtration rate.

## Discussion

This study is the first to clarify the impact of renal function on the association between localized Stage II/III periodontitis and proteinuria in young adults. The principal finding of this study was that localized periodontitis was associated with a higher risk of proteinuria, a marker of acute or chronic kidney injury, in young adults with Stage 2 CKD, whereas the association was null in participants without Stage 2 CKD.

Previous systematic reviews have revealed an association between kidney health and generalized periodontitis in middle-aged or older adults and indicated that periodontitis might contribute to the development of CKD^8,15,22^. According to the biological hypothesis of distant pathogenic effects^23,24^, systemic inflammation is related to generalized periodontitis due to increased exposure time to microorganisms and their products in the blood circulation through persistent bacteremia. Interestingly, elevated levels of IgG directed against periodontal pathogens, such as *Aggregatibacter actinomycetemcomitans*, *Porphyromonas gingivalis*, and *Treponema denticola,* were associated with impaired kidney function^25^. Long-term exposure to the byproducts of periodontitis and its complications might result in disruption of homeostasis, possibly mediating the deterioration of kidney function. In addition, increased periodontitis-related systemic oxidative stress^26^, which shares common risk factors with CKD^12^ is also a possible cause.

Aging affects the progress from localized periodontitis and mildly impaired renal function in young adults to generalized periodontitis and advanced CKD in later life, and this study provides a novel model revealing the association of localized periodontitis with mild CKD in young individuals. The presence of protein molecules in the urine is an early sign of acute kidney injury under stress, for example, surgery^27^, or represents the progressive deterioration of renal function in middle-aged or older individuals^28^. Similarly, acute kidney injury can result in dipstick proteinuria^29^. This study found an association between localized periodontitis and dipstick proteinuria in young adults, specifically in participants with reduced eGFR, highlighting the importance of early treatment of periodontitis in young patients with early CKD. In contrast, the association between periodontitis and proteinuria was null in participants with normal eGFR, probably due to the confounding of exercise-induced proteinuria, which could be considered a benign process in this relatively healthier population^21^.

The participants in the present study were physically fit military personnel and with greater muscle mass. The findings should be interpreted carefully in this study cohort. Participants with eGFR < 90 mL/min/1.73 m^2^ had significantly higher creatinine and blood urea nitrogen levels compared with those with eGFR ≥ 90 mL/min/1.73 m^2^. Some studies had indicated that serum creatinine might not be a good biomarker for measuring kidney function since creatinine is known to be secreted from muscle. In contrast, serum cystatin C is recently reported to be a better biomarker for assessing kidney function instead of serum creatinine^30^. In addition, a greater BMI level in the military personnel, indicating mild or moderate obesity, may reflect greater muscle mass that could not represent an unhealthy status.

This study had some limitations that need to be addressed. First, although we tried to build various models to clarify the effect of kidney function on the association between periodontitis and proteinuria in young adults, strong evidence of causality could not be obtained due to the cross-sectional design and a presence of any considerable bias. Second, information on serum cystatin C and urine microalbumin levels was lacking. Therefore, we could not perform a sensitivity test for comparison. Third, the results should be highlighted that as the AAP/EFP interpreted the extent a bit differently in 2017 and now. Finally, the findings in the military cohort in the present study may not be applicable to the general population of young adults due to the specificity of the military cohort.

## Conclusion

Our findings suggest that among young adults, especially those with a mildly impaired eGFR, localized periodontitis related to systemic inflammation might contribute to acute or chronic kidney injury manifested as proteinuria. The treatment of localized periodontitis in young adults with early CKD might be helpful in preventing acute kidney injury events and deterioration of renal function. Further prospective longitudinal studies are needed for young adults to clarify the association between localized periodontitis, changes in eGFR and the risk of proteinuria.

## Supplementary Information

Supplementary Table 1.

## Data Availability

As the study materials were obtained from the military personnel in Taiwan, the data were confidential and not allowed to be opened in public. If there are any needs for clarification, the readers can contact Dr. Gen-Min Lin, the corresponding author for sharing the data.

## Abbreviations

CHIEF: Cardiorespiratory fitness and health in armed forces study
CKD: Chronic kidney disease
eGFR: Estimated glomerular filtration rate
