# Supplementary material for: Localized periodontitis and kidney function for the risk of proteinuria in young adults in the CHIEF oral health study
Source: Sci Rep. 2022 Nov 8;12:19006. doi: 10.1038/s41598-022-23843-0 (PMC9643363; doi:10.1038/s41598-022-23843-0)
Supplement: Supplementary file 1 — Supplementary Table 1. [file 41598_2022_23843_MOESM1_ESM.docx]

**SUPPLEMENTAL TABLE.** Multivariable linear regression analysis for eGFR and dipstick proteinuria grades with localized periodontitis stages and other periodontal parameters in young adults

|  |  | **Model 1** |  |  |  | **Model 2** |  |  |  | **Model 3** |  |
| --- | --- | --- | --- | --- | --- | --- | --- | --- | --- | --- | --- |
|  | **β** | **95% CI** | ***p*-value** |  | **β** | **95% CI** | ***p*-value** |  | **β** | **95% CI** | ***p*-value** |
| Localized periodontitis stages |  |  |  |  |  |  |  |  |  |  |  |
| eGFR | -0.14 | -0.93 – 0.65 | 0.72 |  | 0.01 | -0.79 – 0.80 | 0.99 |  | 0.01 | -0.79 – 0.80 | 0.98 |
| Proteinuria grades | 0.03 | -0.002 – 0.06 | 0.07 |  | 0.03 | -0.002 – 0.06 | 0.06 |  | 0.03 | -0.002 – 0.06 | 0.07 |
| Probing pocket depth |  |  |  |  |  |  |  |  |  |  |  |
| eGFR | 1.46 | -11.49 – 14.40 | 0.82 |  | 7.57 | -5.67 – 20.82 | 0.26 |  | 8.35 | -4.94 – 21.64 | 0.21 |
| Proteinuria grades | 0.01 | -0.51 – 0.53 | 0.97 |  | 0.01 | -0.53 – 0.55 | 0.97 |  | -0.04 | -0.57 – 0.50 | 0.89 |
| Clinical attachment loss |  |  |  |  |  |  |  |  |  |  |  |
| eGFR | 0.98 | -9.95 – 11.91 | 0.86 |  | 6.19 | -5.00 – 17.38 | 0.27 |  | 6.91 | -4.32 – 18.15 | 0.22 |
| Proteinuria grades | 0.12 | -0.32 – 0.56 | 0.59 |  | 0.13 | -0.33 – 0.58 | 0.58 |  | 0.09 | -0.37 – 0.54 | 0.70 |

Data are presented as **β** and 95% confidence intervals (CI) using multiple linear regression analysis models.

Model 1: age, sex, alcohol intake, smoking and education level adjustments.

Model 2: age, sex, alcohol intake, smoking, education level, BMI, mean blood pressure, fasting glucose, total cholesterol and serum triglycerides.

Model 3: age, sex, alcohol intake, smoking, education level, BMI, mean blood pressure, fasting glucose, total cholesterol and serum triglycerides and remaining teeth.

Abbreviations: eGFR, estimated glomerular filtration rate
